# Maternal plasma miRNAs as biomarkers during mid-pregnancy to predict later spontaneous preterm birth: a pilot study

**DOI:** 10.1038/s41598-017-00713-8

**Published:** 2017-04-11

**Authors:** Clint Gray, Lesley M. McCowan, Rachna Patel, Rennae S. Taylor, Mark H. Vickers

**Affiliations:** 1 Liggins Institute, University of Auckland, Auckland, New Zealand; 2 Department of Obstetrics and Gynaecology, Faculty of Medical and Health Science, University of Auckland, Auckland, New Zealand

## Abstract

More than 10% of babies are born too early resulting in over 15 million preterm births and more than one million new-born deaths globally. Although women with a previous spontaneous preterm birth (SPTB) are considered at high risk for recurrence, the majority occur in women without prior history. Prediction of SPTB risk allows for improved care and potential for targeting novel and existing therapeutics to prevent SPTB, which may result in improved outcomes for infant and mother. In this pilot study, a miRNA array was used to analyse plasma from healthy women in their first pregnancy at 20 weeks of gestation who then went on to deliver either at term or experience SPTB at 28–32 weeks. We identified specific miRNA expression profiles that differentiated between those mothers who delivered at term or delivered following SPTB. miR302b, miR1253 and a clustering of miR548 miRNAs were underexpressed in SPTB cases compared to term controls. Conversely, miR223 was elevated in mothers that later experienced a SPTB. The circulating miRNAs identified in the present study may therefore be attractive candidates as non-invasive biomarkers for the early prediction of SPTB. Further larger studies are now warranted to investigate the potential clinical utility of these markers.

## Introduction

Preterm birth and its complications are estimated to be responsible for 35% of the world’s 3.1 million annual neonatal deaths, and is the second most common cause of death globally after pneumonia in children under 5^1^. Preterm birth also increases the risk of death due to other causes, especially from neonatal infections^2, 3^, and in almost all high-income and middle-income countries, preterm birth is the leading cause of child deaths^1^. Further, a number of those who survive will have a permanent disability. In New Zealand, approximately 8 out of 100 babies are born preterm and globally preterm birth has increased annually by around 2% over the last 20 years^4^. Approximately 60% of all preterm births occur after spontaneous onset of labour and in 40% babies are delivered early because of pregnancy complications. In some settings medically indicated preterm births are responsible for the increase in overall preterm birth over time^5^. Although women with a previous spontaneous preterm birth (SPTB) are considered to be at high risk for recurrence, the majority occur in women without prior history, particularly in women in their first pregnancy^6^. Therefore, accurate prediction of SPTB risk, before the clinical event, would allow for improved care, use of resources, and the potential for targeting novel and existing therapies to prevent preterm birth, which may result in improved outcomes for the infants and their mothers.

Primarily involved in initiating post-transcriptional gene silencing, miRNAs, small noncoding RNAs (19–22 nucleotide sequences long), are known to play many roles in the regulation of gene expression^7–9^. Owing to the wide-spread involvement of miRNAs in the development of and protection from many diseases, there has been increasing interest surrounding their potential as novel biomarkers and therapeutic targets to treat and prevent a number of disease states. There is a rapidly emerging research area investigating the potential of circulating miRNA biomarkers of pregnancy complication and disease with recent work highlighting potential for miRNAs as biomarker of osteoporosis^10^, cancers^11^ and pre-eclampsia^12, 13^. Recent examples have linked alterations in placental and decidua-derived mesenchymal stem cells miRNA expression profiles with pre-eclampsia and suggest that subsets of specific miRNAs may be potential biomarkers for this disease^14–17^. While pre-eclampsia has been a recent focus of miRNA profiling in the area of pregnancy complications, there remains a paucity of data with respect to SPTB. It has recently been shown that distinct cervical miRNA profiles are present in women destined to have a preterm birth^18^. It has also been reported that the levels of specific miRNAs in the human cervix during pregnancy are predictive of gestational age at delivery, and offer potential biomarkers of preterm birth risk^19^. Further work in mice by Mendelson and colleagues has also highlighted specific miRNA clusters that may play a role in parturition via modulation of progesterone receptor activity leading to initiation of labour^20–22^. However, there are few studies that have investigated maternal plasma miRNAs as potential biomarkers of later SPTB risk. Recent work by Elovitz *et al*. examined miRNA blood profiles in women at risk for preterm birth but concluded that profiles were not significantly different in women with preterm birth versus term controls^23^. However, in contrast to the present study, this previous work had examined maternal samples at the time of admission for preterm delivery and as such sampling could not be deemed “predictive”.

In addition, the majority of previous analyses have utilised microarrays and quantitative real-time PCR approaches which lack the sensitivity of digital count technologies such as NanoString^24^. As such, a methodological approach using plasma markers coupled with NanoString high-throughput digital quantification method has yet to be applied in the setting of prediction for those mothers at risk of SPTB. Use of NanoString has several benefits relative to microarray- and PCR-based technologies. NanoString utilises a hybridization method that directly interrogates target sequences via an amplification-free process and thus is highly sensitive, even for low-abundance transcripts such as with plasma miRNA. Thus, this method avoids potential bias that may be introduced via PCR amplification steps. Additionally, measurement is achieved using digital detection of uniquely bar-coded probes, providing absolute quantification. The present study therefore utilised NanoString technology to explore the potential of circulating miRNAs as biomarkers during early pregnancy to predict those individuals that go on to experience a later SPTB.

## Results

### Demographic and clinical findings

There were no significant differences in maternal age, ethnicity, body mass index and primigravida between groups. As expected, gestational age at delivery was significantly different (P < 0.001) between groups (Control, 39.7 ± 1.5 weeks vs. SPTB, 31.4 ± 1.6, p < 0.001). There were no differences in customized birth weight centiles, socioeconomic index score or gestational age at the time of maternal blood sampling (Term control, 20.3 ± 0.73 weeks vs. SPTB 20.0 ± 0.64) (Table 1).

**Table 1:** Demographic and clinical findings.

Variable	Preterm Birth 28–32 weeks n = 7	Controls >39 weeks n = 9	*P*
Ethnicity (% European)*	6 (86%)	8 (89%)	1.0
Age (years)*	29.1 (1.4)	30.8 (0.7)	0.29
Body mass index (kg/m^2^)*	25.9 (1.5)	24.8 (1.3)	0.59
Primagravid*	4 (57%)	6 (67%)	1.0
Socioeconomic index score*	47 (5.4)	48 (3.8)	0.95
Gestation at sampling	20.1 (0.21)	19.9 (0.23)	0.57
Gestation at delivery (weeks)	31.4 (0.6)	39.8 (0.5)	**<0.001**
Customized birthweight centile	58.6 (10.3)	44.2 (7.9)	0.28

For analyses, only those with Nanostring results were included; Term controls; n = 9, SPTB n = 7. Data are number (%) or mean (SEM) as appropriate. *Data are collected at 14–16 weeks’ gestation. P values are for comparing between the two groups using Fisher’s Exact Test or Student t-test. Outside of the gestational age at time of delivery, there were no differences in baseline characteristics between the two maternal groups. There were also no differences between the total original cohort assessed (n = 12 per group) and those for which Nanostring data were subsequently available for analysis.

### Identification of endogenous miRNAs from human maternal plasma

**Figure 1:**
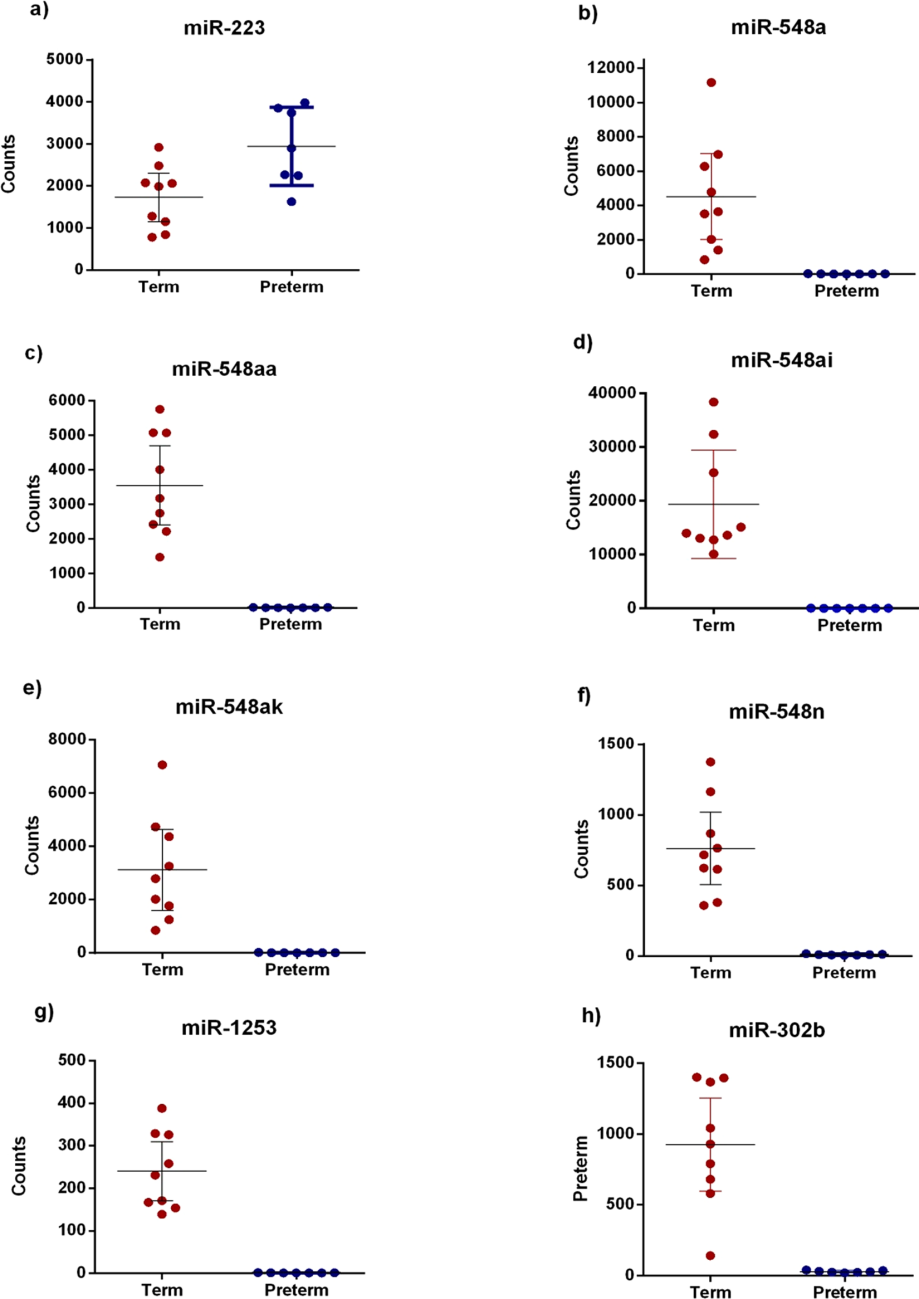
MiRNA profiles in maternal plasma at 20 weeks gestation. Graphs show digital counts of endogenous miRNAs from human maternal plasma at 20 weeks in women who went on to deliver at term or experience SPTB at 28–32 weeks. Individual data are presented along with min-max points with n = 7–9 per group. Data mean +95% CI, p < 0.0001 for all differences for term vs. preterm. Significance was assumed if p < 0.001 by Student t-test.

**Figure 2:**
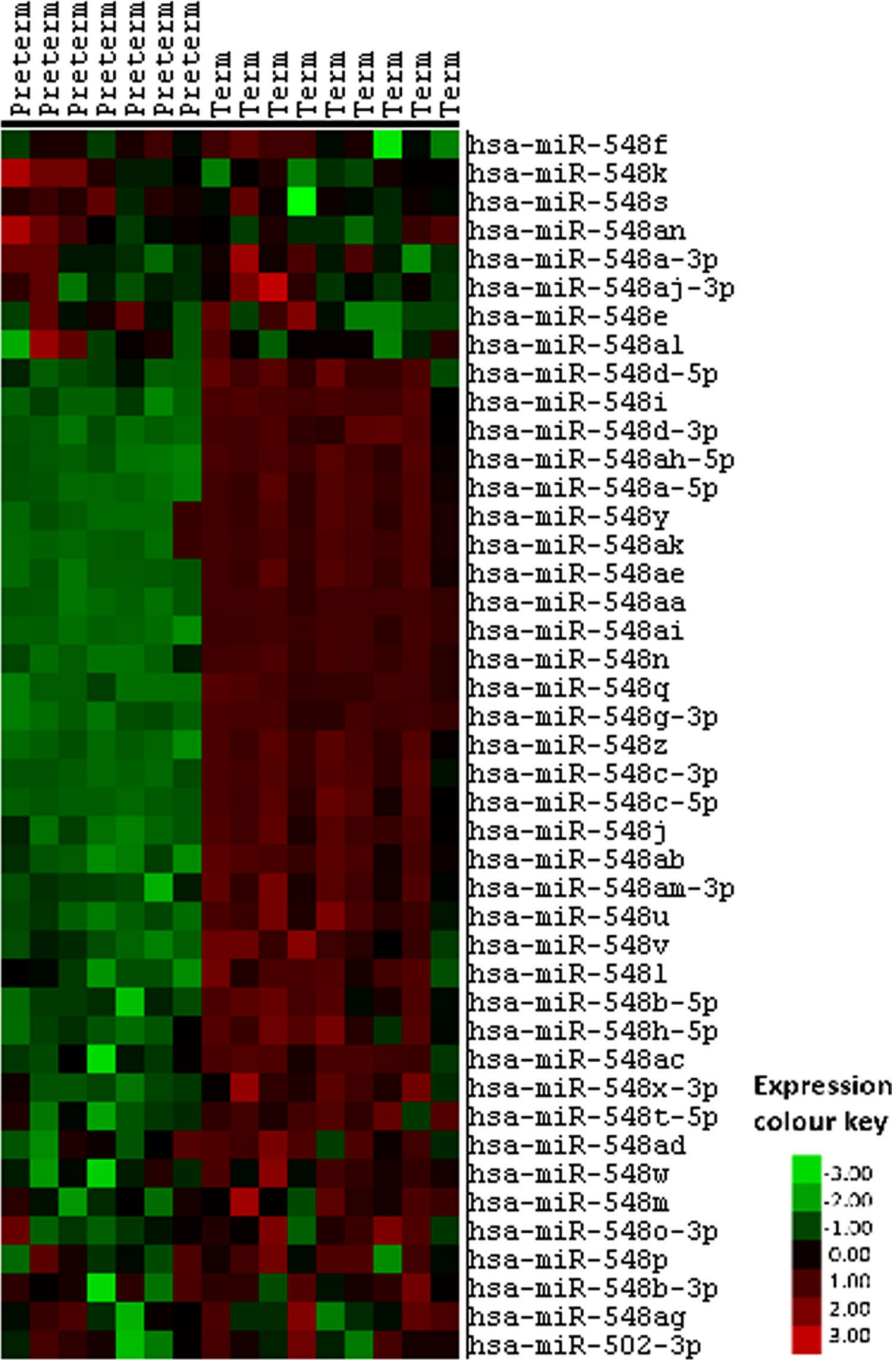
Heat map of endogenous miR548 family expression in maternal plasma at 20 weeks gestation. Heat map shows miR-548 family miRNA from human maternal plasma at 20 weeks gestation in women who went on to deliver at term (n = 9) or experience SPTB at 28–32 weeks (n = 7). Each column represents one plasma sample with red = upregulated, green = downregulated and black = unknown.

miRNA expression profiles were analysed in maternal plasma samples from 24 healthy pregnancies at 20 weeks of gestation of which, 12 went on to deliver at term and 12 had a SPBT (28–32 weeks). Following analysis of 800 human miRNAs, 8 were differentially expressed between term and SPTB groups (Fig. 1a–h) including a cluster around miR548 (Fig. 2).

One miRNA (miR-223), was observed to be differentially expressed between term and preterm birth groups, with miR223 being significantly increased in plasma from the SPTB group (P < 0.001; Control, 1731 ± 223 vs. Preterm, 2945 ± 304, (Fig. 1a)). The remaining 7 miRNAs (miR-548a, miR-548ai, miR-548aa, miR-548ak, miR-548n, miR-1253 and miR-302b) were differentially expressed between groups with all 7 miRNAs being significantly increased in maternal plasma from the term group as compared to the SPTB group (Fig. 1b–h).

## Discussion

Given their stability in blood and, owing to the wide-spread involvement of miRNAs in the development of and protection from many diseases, there has been increasing interest surrounding their potential as novel biomarkers and therapeutic targets to treat and prevent a number of disease states and pregnancy-associated complications^25–29^. Whether changes in maternal miRNA profiles possess utility as a potential biomarker and “fingerprint” for later pregnancy/offspring outcomes is yet to be fully examined. In the current study we have identified distinct miRNAs measured in maternal plasma samples at 20 weeks’ of gestation that are associated with a later SPTB. Using the nCounter NanoString to identify >800 miRNAs in maternal plasma, our results demonstrate that differential expression of plasma miRNA may provide novel non-invasive biomarkers during early pregnancy to identify women at risk of SPTB long before any overt manifestation of preterm complications may occur.

The key miRNAs highlighted in this study include miR223, miR302b miR1253 and a very specific grouping of miR548a, miR548aa, miR548ai, miR548ak and miR548n (Figs 1a–h and 2). Interestingly, of the miR changes we report in this study, only miR223 was increased in the preterm group. All other changes involved a higher expression of miR302b, miR1253, miR548a, miR548aa, miR548ai, miR548ak and miR548n in the term vs. SPTB groups of which, none have previously been associated with preterm birth or placental function. Only one, miR223, of the 8 miRNAs identified has been associated with preterm birth. Our study confirms the previous findings of Hassan *et al*. and Sanders *et al*. in that miR223 had a higher expression in patients that experienced SPTB^19, 30^. However, these studies investigated uterine cervix miRNA expression profiles following term and preterm birth in association to gestational length and not a predictive circulating biomarker miRNA, as in the current study. A further study has examined miRNA blood profiles in women at risk for preterm birth but they concluded that profiles were not significantly different in women with preterm birth versus term controls^23^. Unlike the present study which investigates the potential predictive markers of SPTB in early pregnancy, the work by Elovitz *et al*. examined maternal samples at the time of admission for preterm delivery and as such sampling could not be considered “predictive”.

While miR548a, miR548aa, miR548ai, miR548ak and miR548n have not previously been associated with preterm birth, there remains an overall paucity of information about the function of the miR548 family in disease processes. Downstream targets of the miRNA 548 family have been shown to be involved in breast cancer, inflammatory responses and potential oestrogen receptor sensitivity^19, 23, 31^ and therefore, all have a potential biological role in the setting of preterm birth. Furthermore, miR548 associated target genes have previously been implicated in having a role in breast cancer and immune response^19, 23, 31, 32^. Studies have shown that down-regulation mir548 target genes that have been previously observed to be related to colorectal cancer aetiology. Similar to the current study, these studies showed that down regulation, rather than up-regulation of miR548 was important in the aetiology of disease. Furthermore, a recent paper by Li *et al*. reported a suppression of endogenous miR548 levels in the setting of viral infection^32^. Previous associations with the immune system and colorectal cancer re down regulation of miR548 is in agreement with results presented here, in that a similar down regulation of this miR548 cluster is associated with the incidence of SPTB in the current study. We speculate that the down regulation and their predicted gene associations may indicate that they act through translational repression and may not be repressing pathological mRNA expression changes during the onset and initiation of parturition therefore, allowing a pathological up-regulation of genes aiding the progression of a disease state or, in the case of the current study, initiation of SPTB.

In conclusion, we have identified 7 miRNAs that at 19–21 weeks gestation were elevated in maternal plasma in women with term pregnancies but minimally expressed in those who went on to experience SPTB. We also confirm previous findings that elevated maternal plasma miR-223 expression is associated with SPTB and show the potential that miR-223 may be a viable candidate miRNA biomarker for the early prediction of SPTB risk. Furthermore, a cluster of related miR548s were highly differentially expressed between term and SPTB groups being markedly elevated in 20 week maternal plasma from those mothers that later delivered at term versus those that later experienced a SPTB. It needs to be considered that the change in regulation of the identified miRNAs may reflect a change in maternal immunological status and therefore acting as a sensitive marker of an altered inflammatory phenotype that precedes presentation of more overt symptoms later in pregnancy. Further the assumption cannot be made that the miRNAs identified in the present study are placentally derived. However, it is known that specific placental miRNAs are present in maternal plasma in different ways dependent upon whether the pregnancy is normal or pathological^33^. In the first instance this would require identification of specific miRNAs directly in placental tissue from different disease pathologies and then analysis of maternal blood to assess repeatability and potential utility as biomarkers. Placental tissue was not available for the present study so direct correlations were not possible. To date, limited expression studies have shown that miRNA expression may not be mirrored between the placenta and maternal circulation for certain pathologies while others have shown that miRNAs in blood or in the placenta can increase or decrease in a similar pattern^13, 33, 34^.

Although the low sample size is a significant limitation in the present study, the uniformity of the results obtained does serve to highlight the potential of circulating miRNAs as circulating biomarkers and suggests that specific miRNA profiles may be of clinical importance in the early identification of SPTB. However, due to miRNA levels being extremely difficult to detect in human plasma samples and the increased QC failure of a number of array plates in this preliminary study, larger studies are warranted to confirm the predictive value of these miRNAs in the current study. These novel data suggest that unique circulating miRNA profiles may provide attractive candidates as putative biomarkers for prediction of SPTB risk during early pregnancy. The current pilot study is limited by small sample size and relatively homogeneous subject population and further work in a larger heterogeneous population is now warranted to examine the applicability of the approach in predicting SPTB across a wider gestational age range. This will require an unselected obstetric population comprising of women of different ethnic backgrounds, parity, age, smoking status, BMI and gestational age at sampling and examining the effect of obstetric complications on the utility of the markers identified.

## Methods

### Ethics approval

Informed written consent was obtained from the participants in the SCOPE study, and ethical approval was granted by the Auckland Ethics Committee (AKX/02/00/364). All methods were performed in accordance with the relevant guidelines and regulations of the Auckland Ethics Committee.

### Definition of preterm diagnosis

Normally, a pregnancy usually lasts about 40 weeks. Premature birth occurs before the start of the 37th week of pregnancy. Late preterm is defined as birth, between 34 and 36 weeks of pregnancy, moderately preterm, between 32 and 34 weeks of pregnancy and very preterm, at less than 32 weeks of pregnancy. Extremely preterm is defined as birth at or before 25 weeks of pregnancy. For the purposes of this study our case group involved participants whom went on to experience a very preterm birth between 28 and 32 weeks.

### Study cohort

Using a nested case-control study, maternal EDTA plasma samples at 20 weeks’ gestation were obtained from the New Zealand Cohort of the Screening for Pregnancy Endpoints (SCOPE) study. Cases (n = 12) were participants who had a spontaneous preterm birth resulting in delivery between 28 and 32 weeks gestation. Controls (n = 12) were healthy uncomplicated pregnancies with delivery >=37weeks. They were all non-smokers when they enrolled in the study at 14–16 wks and continued to be non-smokers at the 19–21 week visit, and were matched by ethnicity, age ± 3y, and BMI ± 3 kg/m^2^ (Table 1).

The multicentre SCOPE study was a prospective cohort study of healthy nulliparous women (trial registration ACTRN 12607000551493) with the aim of developing predictive screening tests for preeclampsia, small for gestational age babies and SPTB. The participants were seen at 14–16 and 19–21 weeks of gestation and a detailed interview and clinical measurements were done and blood samples collected. Detailed methods concerning the SCOPE cohort have been previously reported^35, 36^. Customized birthweight centiles were adjusted for maternal height, booking weight and ethnicity, as well as gestational age at birth and sex of infant. Blood was collected in 6 ml sterile plastic EDTA (spray coated) plasma vacutainer BD tubes (Cat. #367873), placed on ice, centrifuged at 2400 g for 10 minutes at 4 °C, transferred into sterile tubes as 0.25 ml aliquots and stored at −80 °C within four hours of collection.

### miRNA isolation and array analysis

RNA was extracted from control term versus preterm EDTA-preserved maternal plasma samples using the miRNeasy® Serum/Plasma﻿ kit (QIAGEN). RNA concentration and purity was estimated spectrophometrically on NanoDrop ND-100 spectrophotometer (NanoDrop Technologies, USA). Digital multiplexed NanoString nCounter analysis system was performed on total miRNA concentrations from each sample. Plasma miRNA samples were then analysed by nCounter Human miRNA panels (800 human miRNA targets) with 6 six internal housekeeping controls. nCounter miRNA Expression Assay kits were used to analyse the digital detection of the plasma miRNAs in a single reaction. Digital detection was performed in two parts; (a) Transcripts detected by probes bound to complimentary segments of DNA which are attached to a unique string of coloured fluorophores. (b) Number of total transcripts in the sample was counted by the number of times is the fluorophore was detected, scanning was performed in 600 fields of view. Nanostring counts represent molecules/100 ng of plasma extract. Following hybridization, counts were analysed by the nCounter Digital Analyzer. The resulting raw data were imported into nSolver (http://www.nanostring.com/products/nSolver) (NanoString Technologies, Inc. Seattle. WA).

### NanoString data analysis

Normalization of raw data was performed using the nSolver software (NanoString Technologies)^24, 25^. NanoString counts were normalised for all target miRNAs by using built-in positive controls. To facilitate statistical analysis, values that were less than <100 molecules in both groups were disregarded and considered not expressed. Thus, given stringent QC criteria, final complete miRNA readouts were obtained for an n = 7 or n = 9 maternal plasma samples for the preterm and term groups respectively. An expression value with a significance value of P < 0.001 was considered statistically significant.

### Statistical analysis

Maternal clinical baseline data was analysed via Students t-test. MicroRNA data was analysed using online miRNA expression software analysis package, NanoString nSolver® platform (NanoString Technologies, Inc. Seattle WA). Data are shown as means ± SEM unless otherwise stated. An expression difference of p < 0.001 was considered statistically significant. All other maternal statistical analysis was undertaken using Sigmaplot 12.0 (Systat Software Inc.).
